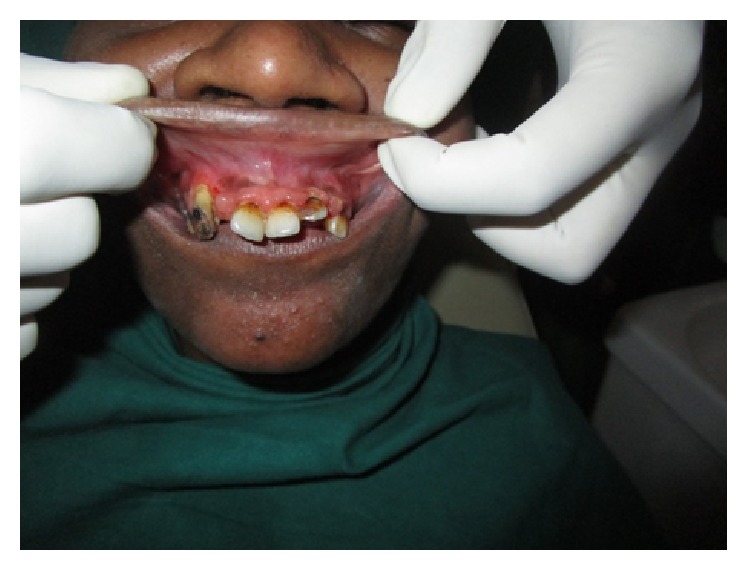# Our Experience in the Management of Traumatic Wound Myiasis: Report of 3 Cases and Review of the Literature

**DOI:** 10.1155/2016/7030925

**Published:** 2016-10-12

**Authors:** Anand Deep Shukla, Abhay T. Kamath, Adarsh Kudva, Deepika Pai, Nilesh Patel

**Affiliations:** ^1^Department of Oral and Maxillofacial Surgery, Manipal College of Dental Sciences, Manipal, India; ^2^Department of Pedodontics, Manipal College of Dental Sciences, Manipal, India

## Abstract

Compromised health and hygiene can lead to many complications and one among them is traumatic wound myiasis. Myiasis is the invasion of living tissues by larvae of flies. Three cases of traumatic orofacial wound myiasis and treatment strategies followed for the management of them are reported in this paper.

## 1. Introduction

Myiasis is a term derived from a Latin word* Muia* which means fly and* iasis* which means disease [1]. The term was christened by Hope in 1849 [2] and explained by Zupmt [3]. It is a pathological condition in which there is growth of dipterous larvae in a living individual which at least for a certain period of time feed on the host's dead or living tissues and develop as parasites [1, 4].

Myiasis is very commonly seen in the rural areas mostly in the animals like cats, dogs, and cows. It is also reported in human beings, especially individuals of low economic status in poor countries [5].

Myiasis can be classified depending on the condition of the tissues that are involved [6]:

*Obligatory*. It requires the presence of living tissues for larvae development.

*Facultative*. It requires the presence of dead tissues for laying eggs and incubating.

 In the human beings the most common site of myiasis is nose, ear, vagina, and skin [7]. It is not very commonly seen in the oral cavity although we report 3 cases of oral myiasis reported to the Department of Oral and Maxillofacial Surgery, MCODS, Manipal, and the different treatment strategies used for the management of them.

## 2. Case Series

### 2.1. Case  1

A 70-year-old male was reported to trauma triage of KMC Manipal with a history of road traffic accident two days back; he was initially admitted in a local hospital for two days, after which he was referred to KMC Manipal. Patient was having a laceration over the chin region which was sutured by the registrar of the Department of Oral and Maxillofacial Surgery. He was admitted under the Department of Neurosurgery as an internal head injury component was present.

**Figure 1:**
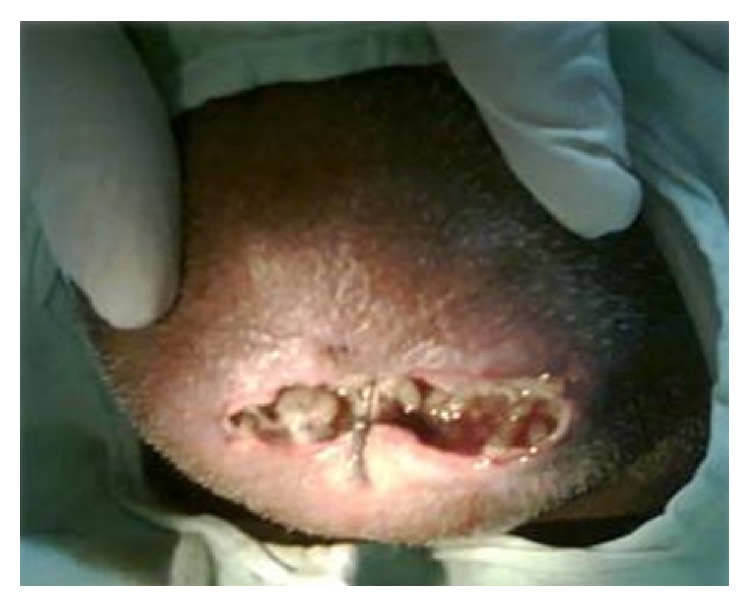


**Figure 2:**
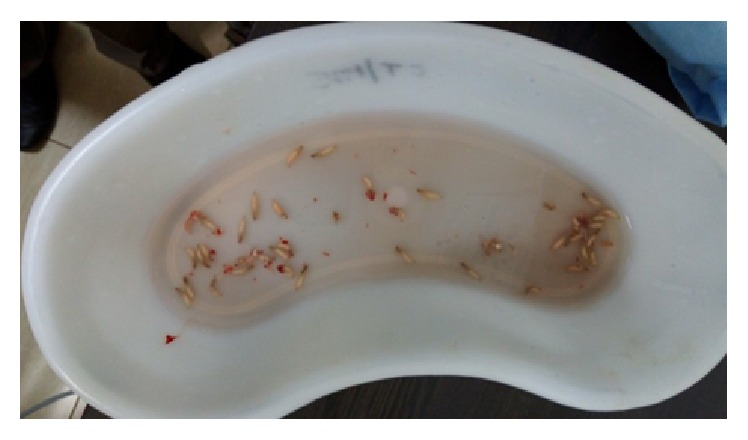


**Figure 3:**
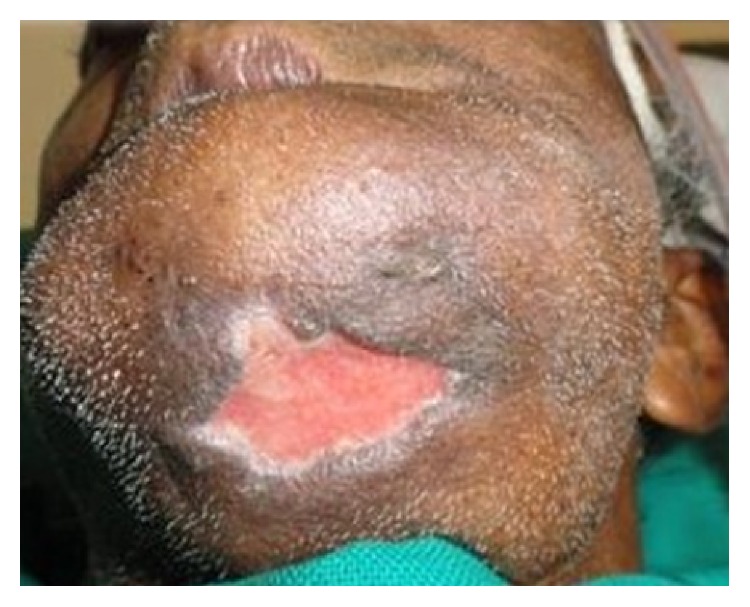


A call was given to us the next day saying that worms were coming out of the sutured chin wound (Figure 1). When the wound was inspected small maggots were coming out of the sutured wound. Once the suture was removed and the wound was inspected small maggots were crawling out of the wound. Around 45–50 live maggots were removed in the ward (Figure 2). Once the accessible maggots were removed, turpentine oil was applied over the wound, and the wound was left open. Daily wound debridement was done and maggots were removed daily over a course of 4-5 days. Topical application of placentrex was also done, which aids in healing of wound. In total 150–200 maggots were removed. Patient also had a left mandibular angle fracture, for which he was taken up for open reduction and internal fixation under general anesthesia. A skin graft was also harvested from the patient's right thigh and secured over the chin wound. Patient recovered well and the wound over the chin region healed without any complications (Figure 3).

### 2.2. Case  2

**Figure 4:**
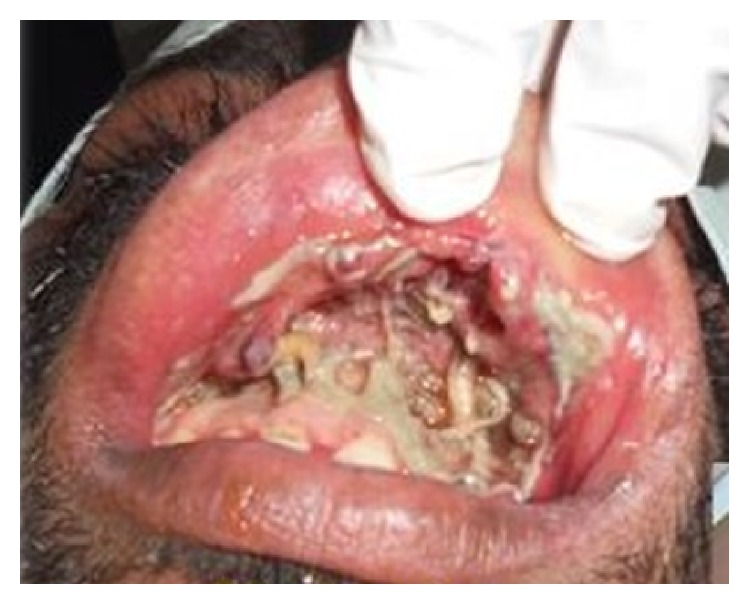


**Figure 5:**
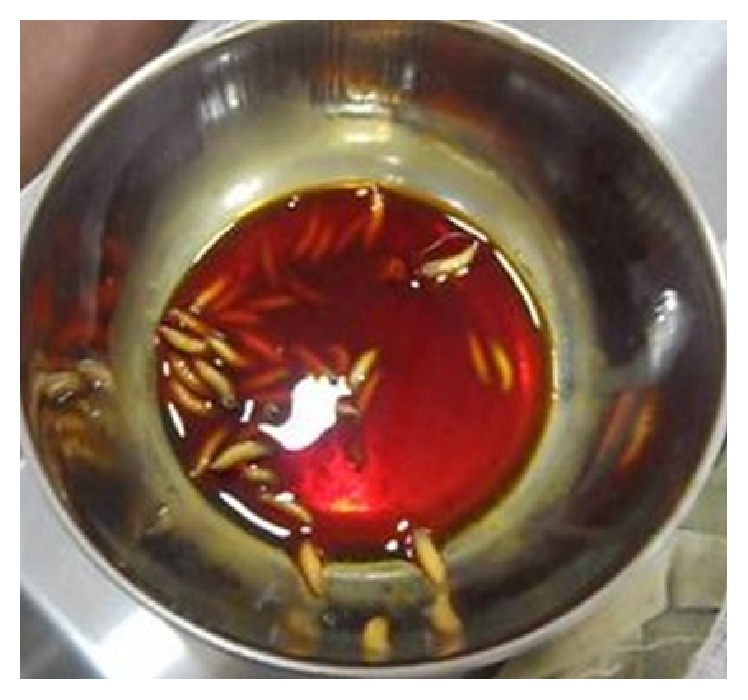


**Figure 6:**
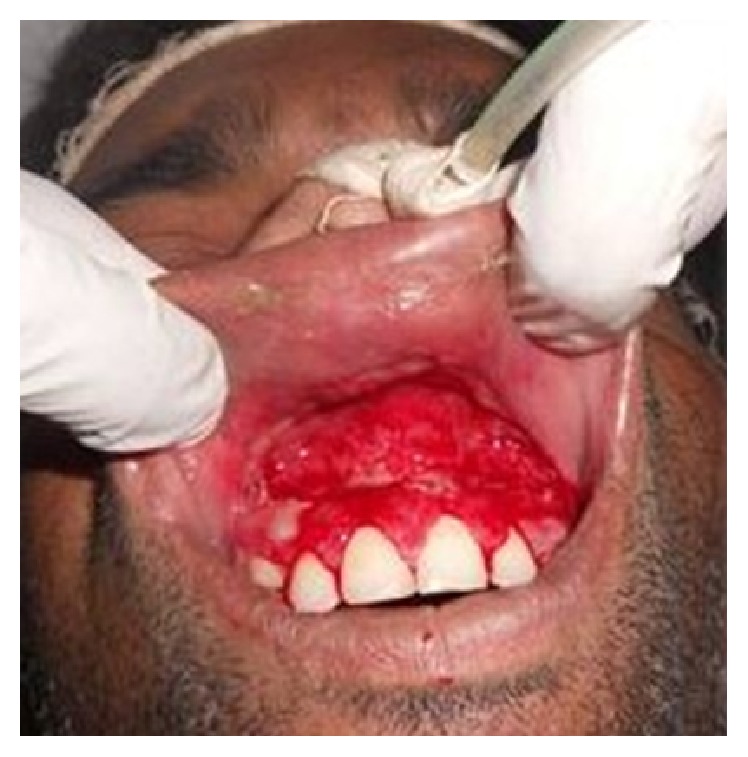


The second case that was reported to us was a 30-year-old known alcoholic male with a history of fall from a tree, following which he was lying unattended there for a long time, when the wound over the intraoral region got contaminated with dust. Patient reported to our OPD one day after the fall with the complaint of pain and swelling over the mouth. Once the wounds were inspected intraorally, live maggots were seen crawling in the patient's oral cavity (Figure 4). Around 35–40 maggots were removed in the OPD (Figure 5) and the patient was admitted for further management of the wounds. Turpentine oil was applied topically over the intraoral wound and maggots that had infested the wound were removed. Daily wound debridement was done and it was supplemented by application of placentrex which aids in granulation. Patient's recovery was uneventful and he showed a very good healing of the wound (Figure 6).

### 2.3. Case  3

**Figure 7:**
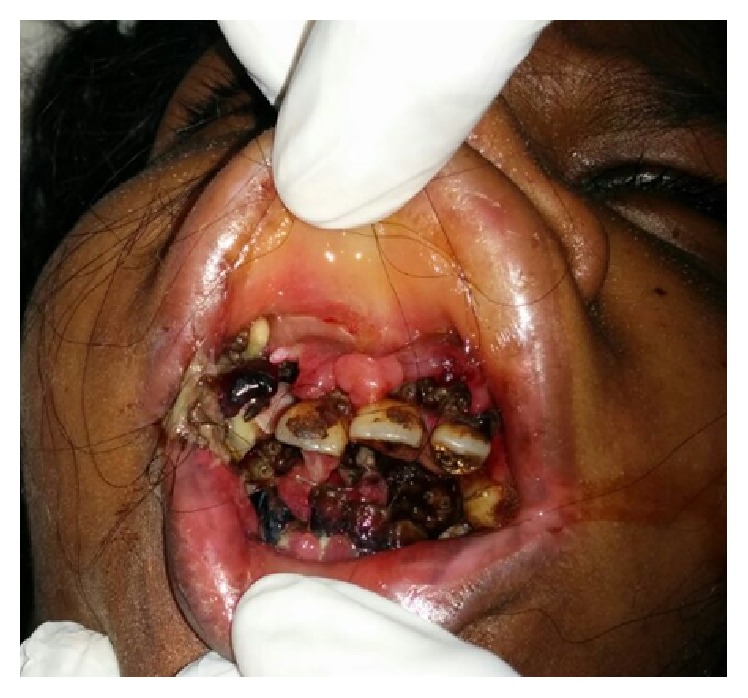


The third case is of a 22-year-old female with mental retardation, who was reported to us with a history of fall at home three days back. Accompanying attendant detailed that she had developed pain and swelling over the mouth since past three days after the fall. On thorough examination she showed presence of live maggots in the oral cavity (Figure 7). The maggots were removed mechanically in the Oral and Maxillofacial Surgery OPD. Around 25–30 maggots were removed, followed by application of turpentine oil. Subsequently patient was admitted for the management of the intraoral wounds and debridement. She was put on IV Antibiotics and analgesics and daily wound debridement was done. She was advised to apply placentrex gel intraorally. Patient recovered well and was discharged after a period of 7 days (Figure 8).

## 3. Discussion

Oral myiasis is most commonly seen in bed ridden patients and patients with special needs who need a caregiver for the maintenance of their oral hygiene [8]. Substance abusers are more likely to suffer from oral myiasis as they are negligent towards their oral and overall hygiene. As can be observed in the presented cases, patients with history of abuse of drugs and alcoholics and bed ridden patients are more likely to present with oral myiasis [9].

A standard guideline for the management of oral myiasis has not been laid down, but the most commonly followed approach is the mechanical removal of all the larvae followed by daily wound debridement and irrigation [10]. Application of turpentine oil over the wound also helps to remove the maggots as it creates an atmosphere deficit in oxygen which forces the maggots to come to the surface, and then they can be mechanically removed [11]. Once the maggots are removed we also subjected the patient to the application of placentrex gel, which aids in faster granulation.

Treatment strategies other than mechanical removal of the maggots include use of larvicidal drugs and occlusion of the area with pressure dressing. Ivermectin has been used to reduce the extent of mechanical debridement of the wound [11]. Occlusion with a pressure dressing reduces the oxygen supply for the maggots causing them to come on the surface, and hence they can be easily removed.

Oral myiasis is more commonly seen in males because of outdoor activities and tendency to neglect the oral hygiene. It is commonly seen in adults, but children can also be affected [12].

In these cases a mechanical removal of the maggots followed by application of turpentine oil was done. Patients were then started on IV Augmentin and Metrogyl. Daily wound debridement was done, supplemented by the application of placentrex gel which aids in faster granulation. In our experience placentrex gel has a very good effect on the healing of oral wounds and should be used more frequently [13].

## 4. Conclusion

Oral myiasis is an uncommon condition seen in the oral cavity. Its incidence can be reduced by raising the quality of life and paying attention to oral hygiene. Old and debilitated patients and patients with special needs are more prone for the development of oral myiasis and hence special care should be taken of these patients. As surgeons it is our duty to raise awareness that a special needs patient should be exposed to proper dental checkup so that complications in the form of oral myiasis do not develop.

## Figures and Tables

**Figure 8:**